# Lyme disease in Australia--still to be proven!

**DOI:** 10.3201/eid0101.950106

**Published:** 1995

**Authors:** R C Russell

**Affiliations:** Department of Medical Entomology, Centre for Infectious Diseases and Microbiology, University of Sydney and Westmead Hospital, Westmead, Australia.


3. Hammerstrom E. Phage typing of *Shigella sonnei*. Acta Med Scand 1949. 133 (Suppl 223).
4. Kallings LO, Lindberg AA, Sjoberg L. Phage typing of *Shigella sonnei*. Arch Immun Ther Exp 1968; 16: 280-7.
5. Frost JA. Testing for resistance to antimicrobial drugs. In: Chart H, ed. Methods in practical laboratory bacteriology. Boca Raton Fla.: CRC Press; 1994.
6. Kapperud G, Rorvik LM, Hasseltvedt V, et al. Outbreak of *Shigella sonnei* infection traced to imported iceberg lettuce. J Clin Microbiol, in press.

### ?Lyme Disease in Australia— Still To Be Proven!

**To the Editor:** The first case of a syndrome consistent with Lyme disease was reported from the Hunter Valley region of New South Wales (NSW) in southeastern Australia in 1982, but there was no confirming serology. More clinical cases, again without serologic confirmation, were reported in 1986, two from the south coast and one from the central coast of NSW. The Queensland State Health Laboratories reported that 186 (14.9%) of 1,247 sera taken from patients between 1986-1989 showed antibody response to *Borrelia burgdorferi* of ≥ 64 by indirect fluorescence antibody test (IFAT), but none of these results were confirmed by immunoblotting.

In 1988, a multidisciplinary investigation of putative Lyme disease began, encompassing clinical, serologic, vector, and reservoir host studies, and results from these studies have been published (1). What follows herein is derived from the accumulated published and unpublished data of the research team, the members of which are credited in the acknowledgments.

Over the past 6 years, principally because of local publicity, there has been an increase in serologic testing for Lyme disease in Australia, particularly in southeastern Australia. Testing has often been initiated by patients with undiagnosed health problems. Thus, most Lyme disease patients seen by infectious disease specialists are self selected and are referred for assessment on the basis of tick exposure and reported positive serologic test results for Lyme disease.

Patients with positive serologic test results frequently have long-standing symptoms for which no other diagnosis has been established. The most common symptoms are musculoskeletal, including myalgias and arthralgias without objective evidence of joint swelling, and syndromes involving fatigue and loss of energy resembling chronic fatigue syndrome. Some patients fulfill diagnostic criteria for fibromyalgia. The next most common symptoms are neurological, and include frequent headaches, inability to concentrate, and memory loss. The most common dermatologic manifestation of chronic Lyme disease, acrodermatitis chronica atrophicans, seen occasionally in Europe and rarely in the United States, has not been reported from Australia.

A few cases of *erythema migrans*, the characteristic dermatologic manifestation of acute Lyme disease, have been reported from southeastern Australia, but clinical diagnosis can be confounded by hypersensitivity reactions to tick bite; a spectacular erythematous reaction is often associated with the bite of *Ixodes holocyclus*, the most common tick biting humans in NSW. Only eight specimens submitted to our laboratory included skin biopsies done to isolate spirochetes. *B. burgdorferi* s.l. was isolated from one patient returning from Europe, but no spirochetes were isolated from local patients.

In our serologic diagnostic service, an enzyme-linked immunosorbent assay (ELISA) for IgG and an IFAT for IgG and IgM have been used with antigens derived from North American *B. burgdorferi* strain B31 (2). From 1988 to April 1994, 78 (1.8%) of 4,372 local patients were positive for IgG by both methods. All 78 patients were tested by IgG Western blot for confirmation by using the virulent North American *B. burgdorferi* strain 297 and a German strain designated B7: with *B. burgdorferi* strain 297, 46 patient samples showed as many as four indicative bands; with the European strain B7, 22 patient samples showed as many as three indicative bands; bands used were 18, 21, 28, 30, 31, 34, 39, 41, 45, 58, 66, 83, and 93 kDa, modified from Dressler et al (3). Twenty-four other patients with various bacterial, viral, or autoimmune syndromes not relating to Lyme disease were tested as controls: with strain 297, 11 control samples showed as many as two indicative bands, and with strain B7, 10 control samples showed as many as two indicative bands.

A high degree of cross-reactivity was demonstrated with the controls, particularly with respect to the 31, 41, 58, and 66 kDa bands for both the European and the American antigen. As none of the 78 patients, including putative late-stage patients positive by ELISA and IFAT, showed more than four specific bands to either antigen, they would be considered negative by the criteria of Dressler et al (3). Fewer than 1% of all referred patients conformed with the national surveillance case definition used in the United States by the Centers for Disease Control and Prevention. Problems of specificity and sensitivity associated with serologic testing for Lyme disease are well recognized, particularly in Australia where no local spirochete has been isolated for use as a reference antigen.

Seroprevalence rates for *B. burgdorferi* infection in humans have been compared between 200 high (rural residents) and 200 low (urban residents) tick exposure groups in coastal NSW, by using the IgG ELISA. No significant difference was found between the two groups, and the overall seropositivity rate was 2.2% (9/400). A parallel survey of dogs in NSW has shown a similar result with an overall seropositivity rate of 2.5% (6/239). These results contrast with those reported from known endemic-disease areas outside Australia that have rural populations with considerably higher seropositive rates. The low rate found by our surveys is similar to that found by other studies undertaken in areas where Lyme disease is not endemic, and humans have 1%-3% positive serologic results cause by cross-reacting antibodies (4).

From January 1990 to December 1992, ticks were collected in areas associated with putative Lyme disease cases and were examined for spirochetes to detect a possible causative agent in potential vectors. Ticks were collected along the east coast of Australia, from southern Queensland through NSW into northern Victoria, by flagging in natural habitats, and from domestic and other native animals. Detection of spirochetes was attempted by dark-field microscopy and culturing of gut contents and by direct testing of ticks using polymerase chain reaction (PCR) to detect the *Borrelia*-specific flagellin gene (5).

In total, more than 12,000 (>1,000 by PCR) ticks were processed, including 7,922 *I. holocyclus* (1). No spirochetes were detected by dark-field microscopy or by PCR. Spirochete-like objects (SLOs), were observed in 94 cultures from bloodfed ticks and only in cultures with bacterial contaminants, presumably from the bloodmeal. Some SLOs yielded positive fluorescence results when tested with *Borrelia*-specific polyclonal antibodies, but tests with monoclonal antibodies (anti-flagellin H9724, anti-OspA H5332, anti-OspB H6831) were negative. Electron micrographs showed that the SLOs were not typical of *Borrelia*, were composed of fibers, and probably were not spirochetes. The electron micrographs were similar to micrographs of SLOs recovered from contaminated cultures from ticks in the United States and Europe and thought to be composed of aggregations of bacterial flagella, probably from the contaminants. Molecular characterization indicated that the SLOs were not related to *B. burgdorferi*.

A small number of native vertebrate animals (13 native rats *Rattus fuscipes*, 3 bandicoots *Perameles nasuta*, and 1 marsupial mouse *Antechinus stuartii*) trapped on the south coast of NSW were sampled by ear-punch biopsy (6) for culture and PCR investigation, but no evidence of borreliae was found. The animal sample was clearly inadequate, and the PCR primers used for the tick and animal studies may have been inappropriate and unable to identify native Australian spirochetes; however the extensive investigations of tick gut contents by culturing and dark field microscopy were also negative for borreliae.

There are some major differences between Australia and the Lyme-disease-endemic areas of the Northern Hemisphere with respect to the natural history of borreliosis. No ticks of the *I. persulcatus* complex, the principal vectors to humans in the northern hemisphere, occur in Australia. In eastern Australia, the logical candidate vector would be *I. holocyclus*, which has a wide host range and is the most common tick biting humans. *I. holocyclus* cannot transmit a North American strain of *B. burgdorferi* (7) but the association with any possible Australian spirochetes remains unresolved. Likewise, none of the mammal species identified as reservoir hosts in the Northern Hemisphere are present in Australia. There are reports of spirochetes in Australian native animals, and a local mammal could be a reservoir host for an indigenous spirochete that occasionally infects humans through a tick vector and produces a clinical syndrome similar to Lyme disease; however, no spirochete was detected in the ticks or animals studied.

The diagnosis of Lyme disease outside known disease-endemic areas should not be based solely on serology because unrelated syndromes, such as autoimmune diseases and cross reactions with other bacteria, can produce false-positive results. Likewise, a definitive diagnosis on clinical grounds alone in a nonendemic-disease area is difficult to justify without adequate scientific support based on isolation of the causative agent from the patient or from another patient or known vector from the region. In Australia, disagreement as to what constitutes a positive serologic result has additionally contributed to overdiagnosis of Lyme disease. Until an organism is isolated from a local patient and is characterized, the presence of Lyme disease in Australia will remain controversial.

### Acknowledgments

My colleagues Rosemary Munro (clinical microbiology), Department of Microbiology, Liverpool Hospital, and Stephen Doggett (entomology), David Dickeson (serology), Danielle Avery (molecular biology), Cheryl Hunt (molecular biology), Joanne Mercer (microbiology), and Nicole Trivett (electron microscopy), CIDM at Westmead Hospital, and John Ellis (molecular biology), Department of Microbiology, University of Technology Sydney, contributed to the studies outlined in this paper. Richard Lawrence, Clinical Superintendent of Medicine, Westmead Hospital, provided valuable discussions on clinical aspects and case presentation. Our investigations were supported by the National Health and Medical Research Council and the Ramaciotti Foundations.

**Richard C. Russell**
Department of Medical Entomology, Centre for Infectious Diseases and Microbiology,
University of Sydney and Westmead Hospital,
Westmead, Australia

### References

1. Russell RC, Doggett SL, Munro R, Ellis J, Avery D, Hunt C, Dickeson D. Lyme disease: a search for the causative agent in ticks in southeastern Australia. Epidemiol Infect 1994;112:375-84.
2. Russell H, Sampson JS, Schmid GP, Wilkinson HW, Plikaytis B. Enzyme-linked immunosorbent assay and direct immunofluorescence assay for Lyme disease. J Infect Dis 1984;149:465-70.
3. Dressler F, Whalen JA, Reinhardt BN, Steere AC. Western blotting in the serodiagnosis of Lyme disease. J Infect Dis 1993;167:392-400.
4. Barbour AG, Fish D. The biological and social phenomenon of Lyme disease. Science 1993;260:1610-6.
5. Persing DH, Telford III SR, Rys PN, Dodge DE, White TJ, Malawista SE, Spielman A. Detection of *Borrelia burgdorferi* DNA in museum specimens of *Ixodes dammini* ticks. Science 1990;249:1420-3.
6. Sinsky RJ, Piesman J. Ear punch biopsy method for detection and isolation of *Borrelia burgdorferi* from rodents. J Clin Microbiol 1989;27:1723-7.
7. Piesman J, Stone BF. Vector competence of the Australian paralysis tick, *Ixodes holocyclus*, for the Lyme disease spirochaete *Borrelia burgdorferi*. Int J Parasitol 1991;21:109-11.

### A Novel Morbillivirus Pneumonia of Horses and its Transmission to Humans

**To the editor:** On September 22 and 23, 1994, veterinary authorities in Queensland and at the CSIRO Australian Animal Health Laboratory were advised of an outbreak of acute respiratory disease in horses at a stable in the Brisbane suburb of Hendra. The trainer of the horses had been hospitalized for a respiratory disease and was in critical condition. At that time, the cause of the horses' illness was unclear and any link between equine and human disease was thought improbable. Poisoning, bacterial, viral, and exotic disease causes were investigated. The history of the horses on this property was considered important (Figure 1). Two weeks before the trainer's illness, on September 7, two horses had been moved to the Hendra stable from a spelling paddock in Cannon Hill (6 km). One of these, a pregnant mare, was sick and died within 2 days. The other horse was subsequently moved on and never became sick. By September 26, 13 horses had died: the mare; 10 other horses in the Hendra stable; one horse, which had very close contact with horses in the Hendra stable, on a neighboring property; and one which had been transported from the stable to another site (150 km). Four Hendra horses and three others (one in an adjacent stable, one moved to Kenilworth, and one to Samford) were later considered to have been exposed and recovered from the illness. Some of these horses were asymptomatic. Nine Hendra horses have remained unaffected. The sick horses were anorexic, depressed, usually febrile (temperature up to 41°C), showed elevated respiratory rates, and became ataxic. Head pressing was occasionally seen, and commonly, a frothy nasal discharge occurred before death.

On September 14, a stablehand at the Hendra stable developed an influenza-like illness characterized by fever and myalgia. The next day, the horse trainer also became ill with similar symptoms. Both had close contact with the dying mare, particularly the trainer who was exposed to nasal discharge while trying to feed her; he had abrasions on his hands and arms. The stablehand, a 40-year-old man, remained ill for 6 weeks and gradually recovered. Besides myalgia, he also had headaches, lethargy, and vertigo. The trainer, a 49-year-old man, was a heavy smoker and showed signs consistent with *Legionella* infection. He ultimately required ventilation for respiratory distress and died after 6 days (Selvey L, et al. A novel morbillivirus infection causing severe respiratory illness in humans and horses, submitted).

At the beginning of the diagnostic investigation in horses, African horse sickness, equine influenza, and hyperacute equine herpes virus were excluded as possible causes by antigen trapping enzyme-linked immunosorbent assay (ELISA), polymerase
